# Mortality and rehospitalization in patients with pre-existing implantable pacemakers undergoing catheter ablation are related to increased comorbidity burden—data from the German Ablation Registry

**DOI:** 10.1007/s00392-024-02449-8

**Published:** 2024-04-15

**Authors:** Gerrit Frommeyer, Florian Reinke, Johannes Brachmann, Thorsten Lewalter, Roland Richard Tilz, Stephan Willems, Florian Straube, Ibrahim Akin, Patrick Lugenbiel, Matthias Hochadel, Jochen Senges, Lars Eckardt

**Affiliations:** 1 Clinic for Cardiology II - Electrophysiology, University of Münster, Albert-Schweitzer Campus 1, 48149 Münster, Germany; 2 Medical School REGIOMED, Coburg, Germany, and University of Split School of Medicine, Split, Croatia; 3 Department of Medicine, Cardiology and Intensive Care, Hospital Munich-Thalkirchen, Munich, Germany; 4 Department of Rhythmology, University Heart Center Lübeck, University Hospital Schleswig-Holstein, Lübeck, Germany; 5 Department of Cardiology, Asklepios Klinik St. Georg, Hamburg, Germany; 6 Department of Cardiology and Internal Intensive Care Medicine, Heart Center Munich-Bogenhausen - Munich Municipal Hospital Group, Munich, Germany; 7 Department of Cardiology, Angiology, Haemostaseology and Medical Intensive Care, University Medical Centre Mannheim, Medical Faculty Mannheim, Heidelberg University, European Center for AngioScience (ECAS), and German Center for Cardiovascular Research (DZHK) Partner Site Heidelberg/Mannheim, Mannheim, Germany; 8 Department of Cardiology, University Hospital Heidelberg, DZHK (German Centre for Cardiovascular Research), Partner Site Heidelberg/Mannheim, Heidelberg University, Heidelberg, Germany; 9 HCR, Heidelberg Center for Heart Rhythm Disorders, University Hospital Heidelberg, Heidelberg, Germany; 10 Stiftung Institut Für Herzinfarktforschung (IHF), Ludwigshafen, Germany

**Keywords:** Cardiac pacemaker, Catheter ablation, Atrial flutter, Atrial fibrillation

## Abstract

**Background:**

Catheter ablation of atrial fibrillation and atrial flutter is routinely performed in patients with implantable devices. The aim of the present study was to assess success rates and potential complications in a large registry cohort of patients with cardiac pacemakers.

**Methods and results:**

The German Ablation Registry is a nationwide, prospective registry with a 1-year follow-up investigating patients who underwent catheter ablation of supraventricular arrhythmias in 51 German centers. The present analysis focussed on the presence of cardiac pacemakers in 591 patients undergoing catheter ablation of atrial fibrillation or atrial flutter. These were compared to 7393 patients without a pacemaker. Patients with pacemakers were significantly older and presented more comorbidities like diabetes, renal failure, cardiovascular disease, or previous stroke. One-year mortality (2.4% vs. 1.3%, *p* = 0.022) and a combined endpoint of death, myocardial infarction, and stroke (3.6% vs. 2.1%, *p* = 0.014) were significantly elevated in patients with pacemakers. Re-hospitalization was also more common in patients with a pacemaker (53.3% vs. 45.0%, *p* < 0.01). After adjustment for important comorbidities, pre-existing pacemaker systems did not show any negative effect. Procedural success was reported in 98.8% vs. 98.4% (*p* = 0.93). Device-related complications were only observed in 0.4% of patients with pacemakers.

**Conclusion:**

Patients with pacemaker systems undergoing catheter ablation of atrial fibrillation or atrial flutter demonstrate an increased risk of death, cardiovascular events, and re-hospitalization. This observation can be largely attributed to an older patient population and an increased rate of comorbidities.

## Introduction

Catheter ablation is frequently employed as first-line therapy of atrial flutter and atrial fibrillation (AF) and is recommended in current guidelines [1]. The combination of atrial flutter and/or fibrillation with electrical device therapy for bradyarrhythmias of various reasons is a frequent finding particularly in older patient cohorts [2]. In these patients, the potential risk of electrode dislocation must be added to other periprocedural risks. Furthermore, the presence of implantable cardiac devices may also serve as an indicator for more severe comorbidities and worse prognosis [3]. Available data does not suggest an increased periprocedural risk of catheter ablation in patients with implanted devices [4]. However, a detailed interrogation of the implantable device should be performed before and after the procedure to ensure appropriate function.

In the present study, data from a multi-center real-world registry on patients undergoing catheter ablation of atrial flutter and atrial fibrillation was analyzed to assess success rates and potential complications in patients with implanted pacemakers.

## Methods

The German Ablation Registry is a nationwide, prospective database on patients who underwent catheter ablation procedures in Germany. Data collection is organized by the *Stiftung Institut für Herzinfarktforschung* Ludwigshafen, Germany (IHF). Fifty-two voluntarily participating German centers committed themselves to include all consecutive consented patients. The local ethics committees approved the registry. Details of the study design and procedures and overall results have been published previously [5–7].

Follow-up was scheduled prospectively at 1 year after catheter ablation by telephone and was conducted centrally by the IHF. During telephone contact, standardized questions on cardiac events (e.g., hospitalizations), complications, medication, and heart failure symptoms were discussed. In case of an ineffective call, further information was gathered from other caring physicians or civil registration offices.

The present study includes patients undergoing ablation of either atrial flutter or atrial fibrillation who previously received a pacemaker system. The cohort of patients who previously received an implanted pacemaker was compared to a control group without implanted pacemakers. Patients with pre-existing or newly implanted ICD or CRT device were excluded. Patients who received AV nodal ablation as therapy of atrial fibrillation were also excluded.

## Statistical analysis

Continuous variables are presented as mean ± standard deviation. Categorical variables are expressed as number and percentage of patients. Differences of categorical distributions were tested for statistical significance using *χ*^2^ tests. For binary variables, odds ratios with 95% confidence intervals were calculated. Rates of rare complications were compared using Fisher’s exact test. The cumulative incidences of death and combined endpoints of death, myocardial infarction, and stroke during follow-up at 366 days after index discharge were assessed using methods of survival analysis (Kaplan–Meier estimator, log-rank test). For adjustment of the difference in 1-year mortality, the following imbalanced baseline variables were included in addition to the existing pacemaker in a forward-selection Cox model: age, sex, coronary artery disease, atrial flutter, NYHA II + , known ejection fraction ≤ 40%, palpitations as main symptom.

*p* values ≤ 0.05 were considered statistically significant. The statistics shown should be regarded as descriptive and were based on the available cases. All calculations were performed using the SAS 9.4 software package (SAS Institute, Cary, NC).

## Results

### Patient characteristics/demographics

Demographic characteristics are summarized in Table 1. Out of 7984 patients registered in the German Ablation Registry between 2007 and 2010, 519 patients already presented an implantable pacemaker at enrollment (Table 1). In total, 72 further patients were either implanted with a pacemaker system or underwent surgical revision of an existing pacemaker system (*n* = 28) during the hospital stay around the catheter ablation while 7393 patients did not have an implantable pacemaker. Of note, patients with implantable pacemakers were significantly older (existing pacemaker: 68.9 ± 9.8 years, newly implanted pacemaker: 72.3 ± 7.3 years) than patients without a pacemaker system (62.5 ± 11.0, *p* < 0.001). Relevant comorbidities including diabetes and coronary artery disease were more common in patients with implanted pacemaker (Table 1).

**Table 1:** Baseline patient characteristics (*LVEF*, left ventricular ejection fraction)

	Existing pacemaker *n* = 591	No pacemaker *n* = 7393	OR (95% CI)
Age (years)	69.3 ± 9.8	62.5 ± 11.0	
CHA_2_DS_2_-Vasc-Score	2.7 ± 1.5	1.9 ± 1.0	
Age > 75 years (%)	24.9	8.8	3.43 (2.80–4.20)
Male (%)	65.0	71.2	0.75 (0.63–0.90)
LVEF < 30% (%)	1.7	2.1	0.82 (0.41–1.61)
LVEF 30–50% (%)	28.7	16.9	1.83 (1.51–2.23)
LVEF > 50% (%)	69.7	81.1	0.59 (0.49–0.69)
Coronary artery disease (%)	37.2	22.2	2.07 (1.74–2.47)
Diabetes (%)	16.4	10.9	1.61 (1.28–2.02)
NYHA 2 + (%)	58.0	45.4	1.66 (1.33–2.06)

### Ablation procedure, discharge, and follow-up

Relevant cardiovascular medication at time of discharge is displayed in Table 2. Patients received catheter ablation for either atrial fibrillation or atrial flutter. Of note, ablation of atrial flutter was more frequently performed in patients with pre-existing pacemakers than ablation of atrial fibrillation. The incidence of first or repeat ablation procedure did not significantly differ between groups. Regarding procedural in-hospital complications, significant differences were neither observed between study groups regarding death, non-fatal myocardial infarction, or non-fatal stroke (Table 3).

**Table 2:** Cardiovascular medication at time of index discharge

	Pacemaker	No pacemaker	OR (95% CI)
ACE-inhibitor/AT1 receptor antagonist (%)	60.1	49.0	1.57 (1.32–1.86)
Betablocker (%)	76.0	73.9	1.12 (0.92–1.36)
Diuretics (%)	45.5	27.2	2.23 (1.86–2.68)
Phenprocoumon (%)	76.6	77.4	0.96 (0.79–1.17)
ASS (%)	21.3	19.4	1.12 (0.92–1.38)
Class I antiarrhythmic drugs (%)	15.2	24.6	0.55 (0.44–0.69)
Class III antiarrhythmic drugs (%)	22.8	17.7	1.38 (1.13–1.69)
Digitalis (%)	7.3	4.4	1.7 (1.23–2.37)
Statin (%)	45.2	31.7	1.78 (1.50–2.11)

**Table 3:** Ablation procedure and discharge (*MI* myocardial infarction)

	Pacemaker	No pacemaker	OR (95% CI)
Atrial flutter (%)	62.9	43.9	2.16 (1.82–2.57)
Atrial fibrillation (%)	37.1	56.1	0.46 (0.39–0.55)
First ablation procedure (%)	84.6	86.3	0.87 (0.69–1.10)
Repeat ablation procedure (%)	15.4	13.7	1.14 (0.91–1.44)
Death (%)	0	0	-
MACE (death, MI) (%)	0	0.1	-
MACCE (death, MI, stroke) (%)	0	0.2	-
Death, MI, stroke, major bleed (%)	0.5	0.7	0.69 (0.22–2.22)

After a follow-up duration of 1 year, patients with implanted pacemakers displayed a significantly increased mortality (2.4%) compared with the control group (1.3%, *p* = 0.022, Table 4). Similar results were obtained for important clinical events including myocardial infarction, stroke, and major bleeding (*p* = 0.02, Table 4). Of note, this finding was based on an increased mortality in patients with an already existing pacemaker while patients with pacemaker implantation during hospital stay did not show an increased risk for events. No significant differences were observed for recurrence of arrhythmias. Reasons for re-hospitalization have been divided into cardiovascular causes and non-cardiovascular causes and were identical in both study groups (72.0% and 28.0%, respectively).

**Table 4:** Follow-up (1 year after discharge; *MI*, myocardial infarction; combined endpoint includes death, MI, stroke and major bleeding)

	Pacemaker	No pacemaker	OR (95% CI)
Mortality (%)	2.4	1.3	1.91 (1.09–3.35)
MACE (death, MI) (%)	2.7	1.5	1.87 (1.11–3.17)
MACCE (death, MI, stroke) (%)	3.6	2.1	1.76 (1.12–2.78)
Combined endpoint (%)	4.3	2.6	1.63 (1.07–2.47)
Repeat ablation (%)	17.3	18.2	1.07 (0.85–1.34)
Re-hospitalization (%)	53.3	45.0	1.40 (1.17–1.67)

### Adjusted analysis

For adjustment of the difference in 1-year mortality, different variables were analyzed. In this analysis, the presence of an implanted pacemaker did not show any negative effects anymore. However, significant effects on 1-year mortality were observed for age (*p* < 0.001), heart failure (NYHA II + , *p* < 0.001), presence of atrial flutter (*p* < 0.001), and reduced left ventricular function (< 40%, *p* = 0.011). No significant effects were observed for gender and presence of coronary artery disease or diabetes (Table 5).

**Table 5:** Adjusted analysis for common risk factors. Adjustment for 1-year mortality: *n* = 105/7811

Variable	Adjusted hazard ratio	95% CI	*p* value
Pacemaker	1.00	0.56–1.77	0.991
Age (per 10 years)	1.80	1.43–2.28	< 0.001
Atrial flutter	3.10	1.84–5.23	< 0.001
NYHA II +	2.18	1.42–3.34	< 0.001
LVEF ≤ 40%	1.93	1.16–3.20	0.011

## Discussion

**Fig. 1:**
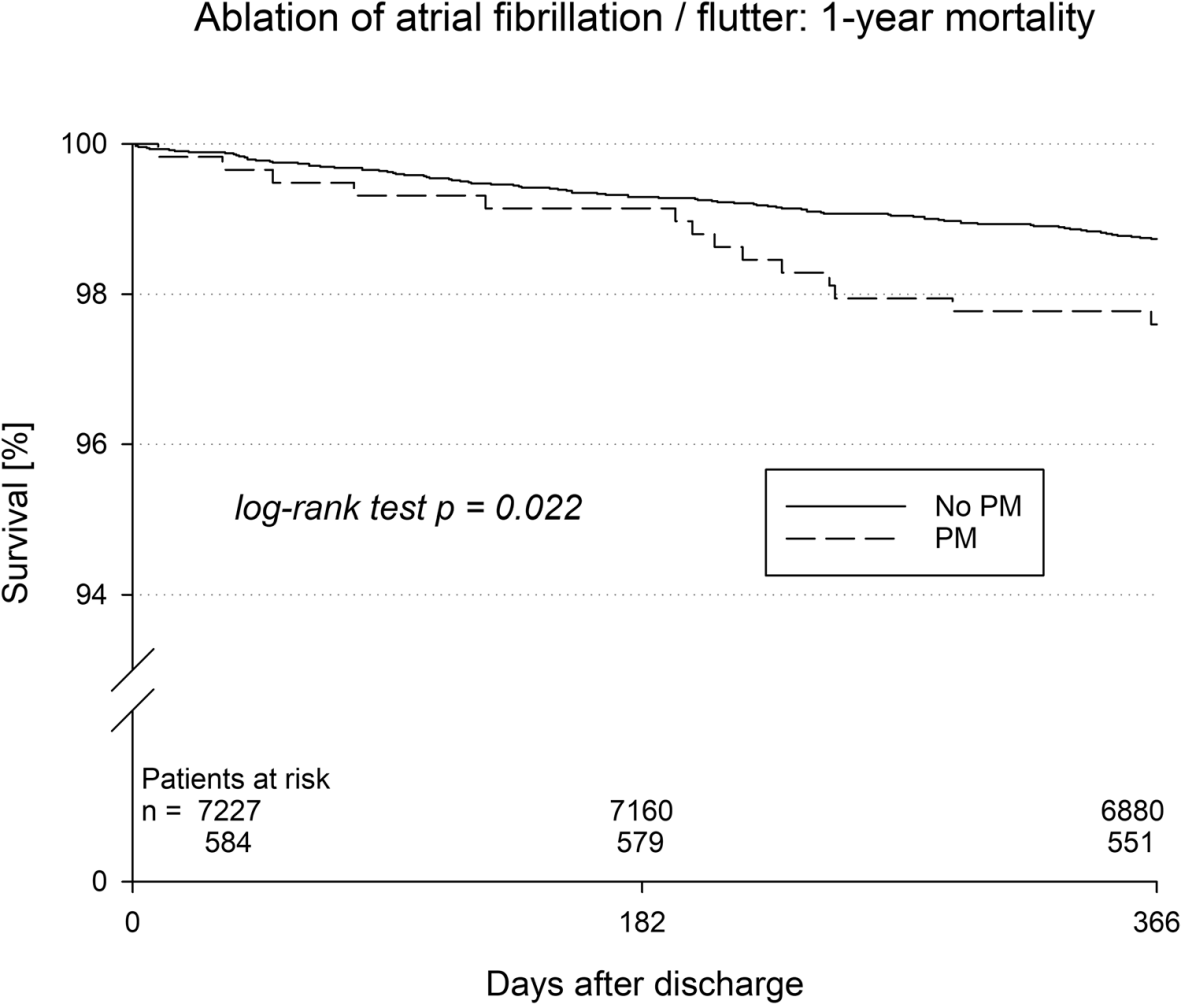
Kaplan–Meier curves for mortality in patients with and without implanted pacemakers

The present study reports “real-world” data on patients with implanted pacemakers undergoing catheter ablation for treatment of atrial fibrillation or atrial flutter. In the presence of increased patient age, the existence of previously implanted pacemakers was associated with an increased mortality and an increased risk of other significant events within 1 year after discharge (Fig. 1).

The present patient cohort is representative for patients undergoing catheter ablation for treatment of atrial fibrillation or atrial flutter. Of note, patients with previously implanted pacemakers were significantly older than individuals without pacemakers. The increased age was accompanied by a higher prevalence of comorbidities such as diabetes or coronary artery disease and, consequently, also by an increased CHA_2_DS_2_-Vasc-Score. Corresponding results have been described in similar patient cohorts [8–11].

### Procedural data and follow-up

Periprocedural complications did not significantly differ between groups. Overall, the incidence of acute severe and of other complications was low. The rate of first ablation procedures and recurrent ablation procedures did not significantly differ, too.

In 0.23% of patients, a surgical revision of the pacemaker system due to malfunction related to the ablation procedure was documented. This underlines the low risk of electrode dislocation during ablation procedures.

During follow-up, patients with a pacemaker demonstrated a significantly increased mortality compared to the control group. Similar results were documented for other severe complications. Furthermore, re-hospitalizations were also more common in patients with implanted pacemakers. These results are most likely explained by demographic aspects and cannot directly be attributed to the existence of pacemaker systems. As described above, patients in the pacemaker cohort were significantly older and presented more relevant comorbidities. The elevated morbidity in the pacemaker group is also mirrored in an increased value of NYHA class.

### Adjusted analysis

After adjustment for certain baseline characteristics, the presence of an implanted pacemaker did not show any negative effects any more. This is easily explained as relevant factors such as increased age, more severe heart failure, or reduced left ventricular function obviously predispose for a worse outcome and in particular for an increased mortality. Therefore, the observed effects in the cohort with pre-existing pacemakers cannot directly be attributed to the pacemakers but to the overall patient cohort and in particular the increased age and higher prevalence of severe comorbidities. Of note, the presence of atrial flutter was also associated with an increased mortality. This observation can be interpreted in line with previously published data where an increased rate of complications was reported in patients undergoing catheter ablation of atrial flutter [12, 13]. This was attributed to an advanced comorbidity profile of the patient cohort, which correlates with the findings of the present study.

## Limitations

The design of the registry may include a selection bias as patient selection is not as objective as in randomized clinical trials. The time of implantation of pre-existing pacemaker systems as well as further details, e.g., the number of implanted leads, was not documented. Likewise, the exact time frame of periprocedural pacemaker implantations or revisions was not reported. Therefore, potential effects of this aspect on the necessity of device revision cannot be interpreted. Furthermore, the analysis of complementary data was not as thorough as in randomized trials. Therefore, the results of the present registry data should be interpreted in an observational and hypothesis-generating way. Lastly, follow-up duration of 1 year is rather short but has been predefined by the design of this registry. Furthermore, data collection was performed between 2007 and 2010. Therefore, the dataset may not be completely representative for current cohorts.

## Conclusion

The results of the present study display “real-life” data on patients with implanted pacemakers who underwent catheter ablation of atrial flutter or atrial fibrillation. The results of the adjusted analysis displayed that the presence of a pacemaker system alone does not increase the risk for a worse outcome. Therefore, a pre-existing pacemaker system especially in elderly patients with atrial fibrillation does not increase the risk of an ablation procedure.
